# Effects of *Moringa oleifera* on Glycaemia and Insulin Levels: A Review of Animal and Human Studies

**DOI:** 10.3390/nu11122907

**Published:** 2019-12-02

**Authors:** Karina Vargas-Sánchez, Edwin Garay-Jaramillo, Rodrigo E. González-Reyes

**Affiliations:** 1 School of Medicine, Universidad de los Andes, Bogotá 111711, Colombia; 2 School of Medicine, Universidad Antonio Nariño, Bogotá 110231, Colombia; edwinmedinv@gmail.com; 3 Universidad del Rosario, Escuela de Medicina y Ciencias de la Salud, GI en Neurociencias-NeURos, Bogotá 111221, Colombia

**Keywords:** *Moringa oleifera*, insulin, diabetes, metabolism

## Abstract

Diabetes and related neurological complications are serious worldwide public health problems. The increasing number of affected individuals make it necessary to implement novel nutritional and therapeutic interventions. The tree *Moringa oleifera* (MO) has been used as a food source and for traditional medicine purposes due to possible antihyperglycemic, antioxidant, anti-inflammatory, and lipid regulating properties. These properties may be explained by the presence of numerous phytochemicals in the leaves, fruits, roots and, oil of the tree. The evidence for acute antihyperglycemic effects of MO extract on diabetic animal models seems to be robust, but more chronic and long-term studies are needed. In contrast, the hypoglycemic effects of MO on humans are not as clear. The scarce number of human studies, together with a diverse range of methodologies and MO doses, may explain this. In addition, evidence regarding changes in insulin levels due to MO intervention is ambiguous, both in animal and human studies. Therefore, more structured studies are needed to clarify if MO has an effect on insulin levels or activity.

## 1. Introduction

*Moringa oleifera* (MO) (also known as “drumstick”) is a tree belonging to the family Moringaceae, genus *Moringa*, originally native from the Himalayans but currently cultivated in many tropical and subtropical regions around the world [1]. Different parts of MO such as leaves, fruits, flowers, and roots have been used as food and in traditional medicine [2]. For example, leaves, the most commonly used part, contain many nutrients including beta-carotene; vitamins B, C, and E; minerals (calcium, iron, potassium, magnesium, etc.); essential and non-essential amino acids; and carbohydrates; among others [3]. MO has been used in traditional medicine for the treatment of various conditions and, more recently, has been proposed to be of benefit in numerous diseases including cardiovascular, diabetes, cancer, neurological, gastroenterological, and inflammatory [4]. Such broad applications attributed to MO, may be explained by the vast amounts of compounds present in the different parts of the tree. For instance, phytochemicals derived from the seeds of MO including glycosidic glucosinolates (GLSs), isothiocyanates (ITCs), nitriles, carbamates, and thiocarbamates, have shown anti-inflammatory, antioxidant, hypotensive, antibacterial, and chemopreventive properties [5].

Metabolic and neurological conditions have a high incidence in the general population and frequently develop into chronic complications, which severely limit quality of life. The present rise in the prevalence of diabetes, metabolic syndrome, and associated disorders (obesity and cardiovascular pathologies) prompts the need for better strategies aimed at improvement of life styles and nutrition, together with the development of more efficient therapeutic alternatives [6]. A major component of metabolic syndrome, hyperglycemia, is a critical factor in the development of diabetes mellitus (DM) and has been related to serious progressive damage in different organs (retina, kidneys, and nerves), as well as to the development of neurodegenerative diseases such as Alzheimer’s disease [7]. DM can cause many different types of peripheral nerve injuries, the most common being a bilateral and symmetric, distal-to-proximal damage to sensory nerves in the feet (in “stocking-glove” pattern), and commonly referred to as diabetic neuropathy. It has been estimated that almost 316 million and 387 million people are affected by pre-diabetes and diabetes, respectively, worldwide, and of these people approximately 200 million develop neuropathy [8].

Different components of MO tree are currently under investigation in order to study possible beneficial effects on the treatment of metabolic conditions, mainly DM. It has been reported in animal models, that MO has antihyperglycemic activity [9] and can improve induced diabetic effects in rats [10]. As well, some studies in vivo and in vitro have observed some positive effects on the nervous system [11]. The main aim of this paper is to review the overall effects of MO on glycaemia and insulin, and to present relevant preclinical and clinical information regarding the use of MO in these aspects. Within this framework, we have focused on animal and human studies of DM, identifying this disease as a potential therapeutic target of MO.

## 2. MO Tree Parts and Overall Mechanisms of Action

### 2.1. MO Fruit, Seed, and Oil

MO seeds, and the oil derived from them, can be used for human consumption an also for commercial purposes [12]. A study on the ethanolic extract of MO seeds revealed the presence of benzyl carbamate, benzyl isothiocyanate, niazimicin, sitosterol, and niazirin [13]. The same publication reported that niazirin showed important antitumoral activities in chemical carcinogenesis. MO seeds have close to 40% oil content, among which is present a high amount of fatty acids, including oleic acid (considered a cholesterol lowering agent), tocopherols, and sterols, among others [14]. Rabbits fed with MO obtained from fruits, together with lovastatin, showed a reduction in the levels of cholesterol, phospholipids, triglycerides, very low-density lipoprotein (VLDL), low-density lipoprotein (LDL), and the atherogenic index, while high-density lipoprotein (HDL) ratio (HDL/HDL-total cholesterol) was increased [15]. In addition, MO possesses more than 30 natural antioxidants in the seeds, including kaempferol, which, apart from antioxidant functions, also improves cellular function and metabolism [16]. As mentioned previously, fruits and seeds contain GLSs, ITCs, nitriles, carbamates, and thiocarbamates, which may confer other potential uses including anti-inflammatory, antihypertensive, antifungal, antiseptic, and due to its high vitamin C content, useful for scurvy prevention [5,14].

### 2.2. Leaves

Due to the presence of both micro- and macronutrients, MO leaves are commonly used as a food source in many regions of the world. The leaves have a high protein content (total protein content 24.8–35.3 g/100 g) together with high levels of calcium, phosphorus, iron, and manganese [17]. Aside from its nutritional properties, MO leaves have also been used in traditional medicine for the treatment of numerous conditions. The leaves are rich in several bioactive compounds including beta-carotene, vitamins (B, C, and E), polyphenols, phenolic acids, alkaloids, GLSs, ITCs, tannins, saponins, oxalates, phytates, and antioxidants [1,18]. Although most studies regarding the effects of substances present in MO leaves have been conducted on animal models, the results obtained suggest a possible benefit for several human conditions. In a recent study, extracts from MO leaves showed antinociceptive and anti-inflammatory activity in a rat model of induced-arthritis [19]. A novel arabinogalactan, *Moringa oleifera* polysaccharide 1 (MOP-1), has been isolated from the leaves of MO showing strong antioxidant properties [20]. In addition, it has also been reported that MO leaves may be of use in viral [21] and bacterial infections [22]. In general, MO leaves have been reported to be of possible benefit for several chronic diseases including cardiovascular conditions, liver diseases, cancer, insulin resistance, and diabetes. For example, cardioprotective effects have been attributed to the presence of quercetin, chlorogenic acid, alkaloids, tannins, ITCs, and B-sitosterol [23].

### 2.3. Roots and Barks

Humans have used both MO roots and bark, mostly for medicinal purposes. Roots possess higher amounts of antinutrients as compared with other parts of the MO tree, limiting its edible use. Roots have higher concentrations of tannins and oxalates, which are not useful as nutritional sources; as well, they contain high levels of carbohydrates, sodium, arginine, lysine, and ascorbic acid (but they lack thiamine, riboflavin, and pyridoxine) [24]. In animal models, the use of bark and roots has proved to serve as an antiulcer agent, together with antisecretory and cytoprotective activity [25]. Other studies have reported various benefits including treatment for poor vision, joint pain, diabetes, anemia, hypertension, toothache, hemorrhoids, and uterine disorders [1].

### 2.4. Mechanisms of Action of MO

Each part of the MO tree provides a mix of nutrients and substances capable of producing a diverse range of effects on the organism. In this section, we will focus on the mechanism of action of the effects of MO extracts on metabolism, mainly on the regulation of glucose.

As mentioned above, several polyphenols are found in MO. Amongst the most important are the flavonoids quercetin and kaempferol, and the phenolic acids chlorogenic acid and caffeoylquinic acid [26]. These compounds seem to confer antihyperglycemic properties, acting as competitive inhibitors of the sodium-glucose linked transporter type 1 (SGLT1) in the mucosa of small intestine (duodenum and jejunum), thus reducing the intestinal absorption of glucose [27]. however, glucose absorption involves other mechanisms such as the glucose transporter 2 (GLUT2), which can be recruited towards small intestine basolateral membrane due to circulating glucose stimulation [28]. In DM, the capacity of the small intestine to uptake glucose is augmented, due to an increase in the expression of GLUT2 and SGLT1 [29]. This produces an extra burden on the patients suffering from DM, further complicated by the fact that most common antidiabetic drugs such as sulfonylureas, biguanides or thiazolidinediones, have primary targets on organs other than the intestines [30]. MO has been studied as an antidiabetic agent due to its effects on the reduction of glucose levels. One of the proposed mechanisms involves quercetin, as this substance can act as an apical inhibitor of GLUT2 [31], although it has no effect on GLUT5 or SGLT1 [32]. Nevertheless, quercetin has also been shown to activate adenosine monophosphate-activated protein kinase (AMPK), to increase glucose uptake through stimulation of GLUT4 in skeletal muscle, and to decrease the production of glucose through downregulation of phosphoenolpyruvate carboxykinase (PEPCK) and glucose-6-phosphatase (G6Pase) in liver [33].

MO aqueous leaf extract has been shown to inhibit the activity of α-glucosidase, pancreatic α-amylase, and intestinal sucrose, contributing to antihyperglycemic properties [34]. These inhibitory effects are possible thanks to phenols, flavonoids, and tannins present in MO. A delay in carbohydrate digestion, caused by the inhibition of these enzymes, leads to a reduction in post-prandial hyperglycemia and hemoglobin A_1C_ (HbA_1C_). These inhibitory effects of flavonoids, including quercetin and kaempferol, have been biochemically explained due to an increase in the number of hydroxyl groups on the B ring, and to the presence of a 2,3-double bond [35]. In addition, these compounds have been studied regarding protective and regenerative properties on pancreatic beta-cells, augmenting insulin production and release [10]. Quercetin induces insulin secretion through phosphorylation of extracellular signal-regulated kinase 1/2 (ERK1/2) pathway together with protection of pancreatic beta-cells against oxidative damage [36].

Leaves from the tree *Juglans regia*, known as common walnut, also possess quercetin and kaempferol. Leaf methanolic extracts obtained from this tree were shown to inhibit the activity of protein tyrosine phosphatase 1B (PTP1B), which is in charge of the negative regulation of insulin-signaling pathway [37]. Furthermore, chlorogenic acid has been observed to enhance glucose uptake in myocytes by inhibition of PTP1B [38]. Moreover, in diabetic rats (but not in the normoglycemic animals), quercetin has been shown to significantly increase the activity of hepatic glucokinase, which mediates the conversion of glucose into glucose-6-phosphate (G6P) and is involved in insulin functional pathway [39]. Chlorogenic acid is also involved in hepatic gluconeogenesis as it can inhibit G6P translocase, decreasing both gluconeogenesis and glycogenolysis [40].

Hyperglycemia can lead to the formation of hydrogen peroxide and ketoaldehydes in the presence of transition metals, which hastens the production of advanced glycation-end products (AGE) [41]. AGE induce many effects including cross-linking of essential proteins, protein function alteration, and cell damage due to reactive oxygen species (ROS), together with downstream activation of proinflammatory pathways like nuclear factor κB (NF-κB) [8]. A high presence of AGE and glycosylated proteins is related to the development of chronic DM complications as retinopathy, nephropathy, neuropathy, and cardiovascular conditions. Polyphenols from a MO aqueous leaf extract have been shown to inhibit protein oxidation, formation of AGE, and protein cross-linking in glycation reactions [42]. Polyphenol protection against protein glycation is believed to be possible thanks to their ability to scavenge free radicals derived from glycoxidation processes [43]. Therefore, several components of MO such as kaempferol, quercetin, chlorogenic acid, gallic acid, and ellagic acid, among others, have been studied as protective agents against ROS and free radical oxidation of DNA, proteins, and lipids [44,45,46]. Moreover, a recent in vitro study shown that treatment with MO reduces acetylation of mitochondrial proteins, increases the amount of supercomplexes and complex I activity, and decreases the activity of uncoupling protein 2 (UCP2), suggesting a possible explanation of its antihyperglycemic effects based on modulation of the mitochondrial respiratory chain [47]. The antioxidant properties of MO extract agents could also prevent the oxidation of lipids, acting as hypolipidaemic and antiatherosclerotic [48].

In addition to its antioxidant properties, extracts obtained from leaves, flowers, and seeds from MO have also exhibited anti-inflammatory effects [49,50,51]. These properties are mainly attributed to the presence of flavonoids, phenolic compounds, and ITCs. It has been shown that some of these compounds provide an anti-inflammatory effect, decreasing the expression of nitric oxide synthase (NOS) and inhibiting the activity of cyclooxigenase-2 (COX-2) [52]. In DM, an overproduction of mitochondrial superoxides is present in many organs, including pancreas, leading to an increase in ROS production and activation of pathogenic pathways. These ROS are responsible for oxidative stress damage to pancreatic beta-cells and insulin resistance. MO extracts reduce oxidative stress and inflammation, blocking NF-κB pathway activation, and thus reducing the expression of inducible nitric oxide synthase (iNOS) and other proinflammatory agents [53]. In addition, treatment with kaempferol has been shown to inhibit cellular apoptosis in pancreatic beta-cells attenuating the activity of caspase-3 and improving the antiapoptotic response trough cAMP/protein kinase A (PKA) and phosphoinositide 3-kinase (PI3K)/Akt signaling pathways [54].

MO has also been considered as a possible hypolipidemic agent. An aqueous leaf extract from MO was reported to have lipid-lowering properties through reduction in the formation of cholesterol micelles and inhibition of pancreatic lipase, pancreatic cholesterol esterase, and bile acid binding [34]. In addition, upregulation of the expression of peroxisome proliferator-activated receptors (PPAR) α1 (PPARα1) and γ (PPARγ) has been observed in rats which received MO seed powder [55]. PPAR are crucial for lipid metabolism, ketogenesis, and cellular energetic homeostasis, and are expressed in several organs including liver, brain, muscle, and heart, among others. As well, PPAR are involved in inflammation, immunity, and glucose regulation [56]. Thus, MO may help to modulate lipid metabolism attenuating chronic adverse effects of pathologies such as DM and atherosclerosis.

## 3. Preclinical Evidence of MO Effects on Glucose and Insulin

MO extracts have been studied for possible hypoglycemic, hypolipidemic, antioxidant, and anti-inflammatory effects. Several animal studies have evaluated MO mechanism of action and function in chronic disease models as DM. In this review, we comprised relevant investigations on animal models regarding the use of MO extracts on diabetes and glucose metabolism, including changes in insulin levels if reported. In total we found 20 publications, 14 studied leaves, four used seeds, one examined the fruit (pods), while another one used leaves, seeds, and stem (Table 1). Fourteen studies were conducted on rats, while five used mice and only one used both rats and mice.

Glucose intolerance has been improved in a study using diabetic male Goto-Kakizaki rats and nondiabetic male Wistar rats treated with MO leaf extract [27]. A significant reduction in glycaemia at 60 and 120 min after glucose administration was found in Goto-Kakizaki rats as opposed to control Wistar rats, which had a nonsignificant reduction. A study performed on male Wistar rats with diabetes induced by streptozotocin (STZ) intraperitoneal injection (i.p.) (55 mg/kg), showed that treatment with MO aqueous leaf extract at different doses improved glucose tolerance in diabetic rats, as well as reducing glycaemia in controls, and normalizing glycaemia in sub, mild, and severely diabetic rats [60]. The same study used glipizide (a sulfonylurea used to treat DM in humans) as a positive control, finding that the MO extract produced better results than this drug. In addition, the study also observed an improvement in hemoglobin and total protein levels in MO-treated animals and absence of proteins and glucose in urine. Wistar rats of both sexes with diabetes induced after STZ injection (50 mg/kg, i.p.), were treated with a methanolic extract from MO fruits [73]. The authors showed that diabetic animals treated with MO presented a reduction in blood glucose and nitric oxide, together with an increase in serum insulin, protein levels, and improved antioxidant function. The same study used glibenclamide (a sulfonylurea commonly used to treat DM in humans) as a positive control, and obtained similar results with the animals treated with MO. A similar study with STZ-induced (90 mg/kg, i.p.) diabetes in Long Evan rats showed better performance of MO ethanolic leaf extract as compared with glibenclamide, although MO-treated diabetic animals showed no changes in insulin levels [9]. A recent publication reproduced these results obtaining comparable results from MO-treated diabetic Sprague-Dawley rats and glibenclamide-treated diabetic Sprague-Dawley rats [57].

Female alloxan-induced (100 mg/kg, i.p.) diabetic Wistar rats treated with MO aqueous leaf extract, had a significant glycaemia reduction, returning to normal levels, together with improvement of lipid metabolism [10]. Furthermore, treatment with MO provided evidence of hepatocyte and pancreatic beta-cell regeneration. A significant glycaemia reduction was observed in STZ-induced (60 mg/kg, i.p.) diabetes male Sprague-Dawley rats treated with MO aqueous leaf extract [61]. This study also showed that MO treatment preserved the histology of beta-cells, increased glutathione (GSH), and reduced malondialdehyde (MDA). Rats with STZ-induced diabetes (60 mg/kg intravenous (i.v.)), were treated with either 50 or 100 mg/kg of MO seed powder, finding a significant reduction in glycaemia, but failing to return to control levels [69]. In addition, the authors reported that treatment with MO improved various factors including lipid peroxidation, antioxidant enzymes (MDA, catalase, superoxide dismutase (SOD), and GSH), immunoglobulins (decreased IgG and IgA), anti-inflammatory (decreased IL-6), HbA_1C_, electrolytes (sodium and potassium), and urinary and kidney functions (creatinine, blood urea nitrogen (BUN), and uric acid). Although both treatments with MO (50 and 100 mg/kg) significantly improved the previously mentioned factors, the dose of 100 mg/kg was more effective. Similar antioxidant and hepatoprotective results were observed in other alloxan-induced and STZ-induced diabetes rat models [62,65]. In addition to its antihyperglycemic function, MO extract have also shown mitochondrial protective effects. Treatment with MO methanolic leaf extract on STZ-induced (55 mg/kg, i.p.) diabetic Wistar rats, showed elevation of heme oxygenase-1 and GSH levels and a reduction in ROS formation and in lipoperoxidation in liver mitochondria [66]. Another study, using male Wistar rats with alloxan-induced diabetes (120 mg/kg, i.p.) and treated with MO methanolic leaf extract, showed a significant reduction in glycaemia and a significant increase in insulin levels as compared with non-treated diabetic animals [64]. The authors found that treatment with MO produced similar results to treatment with metformin (a biguanide frequently used to treat DM in humans). Furthermore, the same study observed a significant reduction in blood total cholesterol, LDL, and triglycerides, and a significant increase in HDL. The effects of MO have also been studied in diabetic transgenic mice models. Tang et al. [68] treated C57BLKS/J Iar-+Lepr^db^/+Ledpr^db^ and C57BLKS/J Iar-m+/Lepr^db^ mice with a MO ethanolic extract obtained from leaves, seeds, and stem, and found a significant decrease in blood glucose concentration from day 14 to day 35, as well as a significant increase in insulin levels and a significant decrease in triglycerides and LDL. The ethanolic leaves extract showed the strongest antioxidant effects as compared with seeds and stem, but glycemic effects were only studied on leaves.

Olurishe et al. [67] showed that chronic administration (up to 42 days) of MO ethanolic leaf extract together with sitagliptin (an oral antihyperglycemic used in DM type 2) were useful for glycemic reduction in alloxan-induced (150 mg/kg, i.p.) diabetic Wistar rats, although administration of MO alone only produced a significant reduction in fasting blood glucose levels on day 21, failing to offer a significant reduction at later times of the study. Furthermore, the same paper reported no significant reduction in insulin levels with MO extract treatment, as well as no changes in hyperglycemic induced pathologic lesions in the retina. A related study by the same group, also using alloxan-induced diabetic Wistar rats treated with sitagliptin and MO ethanolic leaf extract, reported no changes in the levels of blood electrolytes or albumin (glycaemia or insulin were not measured), a significant increase in urea levels and triglycerides, and no benefit over kidney function or structure [74]. A recent study done in STZ-induced (45 mg/kg, i.p.) diabetic female Wistar rats and in high-fat diet induced diabetes female C57BL/6 mice, showed both acute and chronic significant reduction in glycaemia in the animals treated with MO aqueous leaf extract [63]. The chronic effects were only measured up to day 22, so it is unknown if this study could have shown similar results to those by Olurishe et al. [67] that measured glycaemia up to 42 days. In addition, both diabetic rats and mice treated with MO presented improvement of hepatic functions, a significant reduction in total cholesterol, triglycerides, LDL, and VLDL, and a significant increase in HDL. The MO aqueous leaf extract effects were more effective on the diabetic mice model than the rat diabetes model. A study that induced obesity and insulin resistance after feeding five-week-old C57BL/6J mice with a very-high-fat diet, showed MO treatment improved glucose tolerance [70]. Animals were given isothiocyanate-enriched MO seed treatment mixed with either very-high-fat or low-fat diets ad libitum up to 12 weeks. The study reported that MO-treated mice had reduced blood glucose as compared with control (very-high-fat diet without MO treatment) mice, during the 12 weeks. The low-fat diet animals only showed a blood glucose reduction during week nine of the study. In addition, the authors reported other benefits of the MO treatment, such as anti-inflammatory, antioxidant, and modulation of gut microbiome.

Specific compounds from the MO tree, including N,N′-bis{4-[(α-L-rhamnosyloxy)benzyl]}thiourea, S-Methyl-N-{4-[(α-L-rhamnosyloxy)benzyl]}thiocarbamate, and niazirinA, have also been studied for possible antihyperglycemic and antidiabetic effects in preclinical experiments. A recent study found that the above compounds, obtained from MO seeds, significantly reduced blood glucose levels in STZ-induced (40 mg/kg) diabetic Institute of Cancer Research (ICR) mice [71]. A protein isolate obtained from MO leaves (57.5% of the soluble protein of the leaf extract) was studied in alloxan-induced (150 mg/kg, i.p.) male diabetic mice [59]. A single i.p. dose of the protein isolate at 300 and 500 mg/kg (but not with 100 mg/kg) was able to induce a significant hypoglycemic reaction in the diabetic mice, although, after heating the protein isolate at 98 °C or after oral administration, the effect was abolished. This protein isolate failed to alter insulin or SOD levels, but significantly increased catalase and reduced MDA levels.

A metabolic syndrome model induced through a high-fat diet in Wistar rats showed that a three week MO leaf powder treatment (700 mg/Kg once per day) was able to improve results on the oral glucose tolerance test, together with a decrease in triglyceride levels and in abdominal circumference [58]. No changes were observed in insulin tolerance test, total cholesterol, blood pressure, or fasting glucose.

The previous investigations showed a hypoglycemic effect of MO in different rodent models under various conditions. Despite the use of a very wide range of MO doses, strongest antihyperglycemic effects on diabetic animals were mostly reported between 200 and 300 mg/kg. The study by Wang et al. [71], showed significant hypoglycemic effect with a low value of 20 mg/kg, although this result may be explained as this study used isolated MO compounds, instead of tree parts, so the doses are not comparable. As mentioned previously, several components of MO may explain these effects on glycemia. For example, the flavonoid kaempferol has been shown to improve glycolysis, glucose uptake, glycogen synthesis, AMP-activated protein kinase (AMPK) activity, and GLUT-4 expression [58,75].

Most papers found, failed to report effects of MO on insulin levels. As a critical component of DM, future studies should evaluate insulin levels together with glycaemia levels in order to determine an overall effect of MO on this disease. Evidence for insulin-modifying effects of MO is still under scrutiny. The publications of Gupta et al. [73], Olayaki et al. [64], and Tang et al. [68], reported an increase in insulin in diabetic rodent models treated with MO methanolic fruit, methanolic leaf, and ethanolic leaf extracts, respectively. While a decrease in insulin levels was reported in a Wistar rat model for polycystic ovary syndrome treated with MO aqueous leaf extract, although this last study failed to measure glycemic levels [76]. Furthermore, no changes in insulin were reported in the works by Azad et al. [9], Olurishe et al. [67], Paula et al. [59], and López et al. [58]. These variations in obtained results regarding insulin may be explained by the diverse experimental conditions in the studies, where tree parts, animal model, and MO doses were different (Table 1).

## 4. Clinical Evidence of MO Effects on Glucose and Insulin

Despite the number of in vitro and animal studies reporting the antihyperglycemic effects of MO, few clinical trials or studies have been conducted in humans. In our search, we found six published studies, which measured the effects of MO on blood glucose levels in humans (Table 2).

A study conducted on postmenopausal women without diabetes, showed that daily oral consumption of 7 g of MO leaf powder during three months, significantly reduced fasting blood glucose levels and increased hemoglobin values [77]. In addition, several antioxidant markers were modified in these women, reporting a significant augment in serum retinol, serum ascorbic acid, glutathione peroxidase, and SOD, whereas a significant decrease of MDA was observed. The authors of this publication mentioned no secondary or negative effects after MO ingestion. Although, the authors failed to report if background diet was either controlled or monitored, therefore, is unclear if other dietary or external factors affected the results. A study conducted on male and female healthy volunteers receiving increasing oral doses of MO leaf powder (1 g, 2 g, and 4 g) every two weeks, showed no significant reduction in fasting plasma glucose, but a significant increase in insulin blood level [78]. The largest increase in insulin was found when the subjects received 4 g of MO leaf powder. In addition, the authors reported no changes in BUN, creatinine, alanine aminotransferase (ALT), and aspartate aminotransferase (AST) after MO ingestion. No secondary or negative effects after MO ingestion were reported. The authors failed to mention if background diet either was controlled or monitored, thus, is not clear if insulin changes observed were due to an external factor. Another study done on male and female healthy volunteers which received a single oral dose of 500 mg MO aqueous leaf extract, showed no significant changes in the levels of fasting plasma glucose at 30, 60, 90, and 120 min after MO ingestion [79]. However, acute changes were observed in other aspects, reporting a significant decrease in MDA levels and a significant increase in antioxidant markers. Participants were asked to fast for at least 8 h before MO ingestion and to maintain their typical diet, physical activity, and general lifestyle throughout the study. Furthermore, a randomized placebo controlled clinical study that compared the effect of MO leaf capsules and placebo in therapy-naïve type 2 DM patients, found no significant differences in glycaemia or HbA_1C_ levels [80]. The patients received 4 g of MO leaf powder capsules twice a day, before breakfast and before dinner, over a period of four weeks. No incidence of hypoglycemia presented with MO treatment, although four patients reported transient diarrhea. As well, no significant differences were found for BUN, creatinine, ALT, and AST. The authors failed to mention if background diet either was controlled or monitored. Another study examined the effects of 12 week MO treatment (500 mg three times/day) in DM patients, finding significant plasmatic reductions in both HbA_1C_ levels and high specificity C-reactive protein [81], however, this study has serious limitations, which included not reporting the changes in blood glucose or insulin, no randomization or blinding in study design, not specifying the type of diabetes of participants, and having a very large age distribution, among others. Each participant had a home partner responsible for monitoring the compliance of the treatment, as well as to prevent smoking, alcoholic beverage intake, and endurance exercise a day before the blood extractions. Finally, a recent study conducted on type 2 DM and healthy volunteers from a Saharawi population living in refugee camps, shown a significant reduction in glycaemia and in α-amylase activity after MO ingestion [82]. Participants were provided a meal supplemented with 20 g of MO leaf powder, and glycaemia was determined postprandial at 30, 60, 90, 120, 150, and 180 min. No significant differences in glycaemia were observed for the healthy subjects, while significant reductions in glycaemia were observed at 90, 120, and 150 min after MO treatment in individuals with diabetes. The researchers prepared a specific meal for the participants, based on 80 g of rice and 160 g of camel meat stew. Therefore, the diet was tightly controlled and homogenous between all participants. Insulin or HbA_1C_ levels were not reported.

Two studies (NCT03189407 and NCT02308683) involving MO and diabetes are registered in the United States National Institute of Health (NIH) clinical trials database, https://clinicaltrials.gov/. The status for both studies appears as completed but no research results are provided. Study NCT03189407 was done on type 2 DM patients receiving metformin together with 400 g of MO in tea twice a day. This study planned to measure blood glucose levels and changes in plasmatic levels of metformin. Study NCT02308683 was done on adult DM patients, which received a 12 week supplement of MO and corresponds to the publication [81], included in Table 2.

## 5. Conclusions

The tree MO has been proposed and studied as an antidiabetic agent. In this review, we included animal in vivo and human reports about the role MO may play in diabetes, focused on changes in glycaemia and insulin.

Ingestion or treatment with MO induces robust changes in glycaemia levels. All animal studies included in this review reported a significant reduction either in glycaemia or in glucose tolerance tests. Regardless of the tree part used (leaves, seeds or fruit), or the type of extract (powder, aqueous, methanolic or ethanolic), all diabetes animal models showed a significant reduction of glycaemia in diabetic animals treated with MO as compared with diabetic controls. The evidence for acute antihyperglycemic effects of MO extract on diabetic animal models seems to be robust, but more chronic and long-term studies are needed. This is important, as DM and metabolic syndrome have a chronic nature and novel therapeutic (or complementary) agents need to take into consideration this aspect. An important limitation regarding the studies included in this review is related to the wide variation in the parameters reported. In particular, the absence of other supporting measurements of glucose metabolism, such as glycosylated hemoglobin or glucagon, limits the conclusions regarding the effectiveness of MO treatment in animal models of diabetes. Therefore, future research should examine not only hypoglycemic effects of MO, but also additional parameters related to glucose function.

Opposed to animal studies, only two studies in humans, one conducted on postmenopausal women and one in type 2 DM, showed a significant glycaemia reduction, the other four, two with healthy subjects and two in patients with DM, failed to show a significant reduction. These results are difficult to compare as all studies had different methodologies and used a diverse range of doses. For example, the study on postmenopausal women used the highest amount of MO and for a longer period (7 g daily for three months) as compared with the other five studies. Similar to healthy human subjects, many animal studies failed to observe a significant decrease in glycaemia after MO use in nondiabetic controls, suggesting that glucose regulation may be more effective under pathological conditions. A more precise isolation and characterization of the various phytochemicals present in MO may be of help determining which ones (or a combination of) produce acute or chronic glycaemia lowering effects. Nevertheless, the evidence indicates that the ingestion of MO is safe both in animals (between 50 and 700 mg/kg) and in humans (between 500 mg to 7 g). This safety profile suggests the design of nutritional investigations including MO as a supplement or complement for patients suffering from metabolic or even neurological diseases. Despite the safety profile of MO, a possible limitation in human studies, in particular long-lasting trials, may be related to the taste of MO, as poor taste acceptability of this tree in meals has been reported [82]. In addition, most studies used MO leaves, and only a few were done with seeds or fruits. Therefore, more comparative studies between the different parts of the tree are also suggested in order to determine which tree part (or a mix of them) is more efficient.

Evidence regarding changes in insulin levels due to MO intervention is not as robust as that for antihyperglycemic effects. Some works showed a significant increase in insulin levels, but many other studies on rats and mice failed to replicate this. Similar findings occurred in human studies, where only one (in healthy subjects) reported insulin, showing a significant increase in insulin levels in individuals treated with MO. Therefore, more research is needed to clarify if MO has an effect on insulin levels or activity. This is important when determining the type of DM on which intervention is planned, as type I would benefit from insulin increase, and type II, due to hyperinsulinemia, may not. Other effects commonly reported in the animal models and in human studies are in line with in vitro observations such as strong antioxidant and lipid metabolic regulating properties, which could also be of benefit for the treatment of various metabolic and neurological conditions, including diabetes.

## Figures and Tables

**Table 1:** Evidence of the effects of *Moringa oleifera* (MO) treatment in diabetes animal models.

Animal Model	MO Tree Part	MO Treatment	Results of MO Treatment			Ref
			Blood Glucose	Insulin Levels	Other Effects	
Goto-Kakizaki (GK) diabetic rats and nondiabetic Wistar rats used as controls	Leaf powder (in a glucose solution).	Treatment time: 120 min Con: glucose 2 g/kg MO: glucose 2 g/kg + 200 mg/kg MO	MO decreased BG at 20, 30, 45, and 60 min (*p* < 0.05) as compared with controls.	Not measured	Food was retained for longer periods in the MO treated animals.	[27]
Alloxan-induced diabetic Sprague-Dawley rats	Leaf powder,	Treatment time: 8 weeks Con: Untreated Con MO: 50 mg/kg MO Diabetic Con: Untreated Diabetic MO: 50 mg/kg MO Diabetic Exp: 50 mg/kg MO + glibenclamide 600 µg/Kg	At the second week, a significant reduction was observed in BG in diabetic rats treated with MO, from 300 mg/dL to 100 mg/dL as compared with controls.	Not measured	No change in enumeration of lactic acid bacteria.	[57]
Wistar rats with metabolic syndrome (MS) induced with high-fat diet	Leaf powder	Treatment time: 3 weeks Con: Untreated Preventive: 700 mg/Kg/day MO for 3 weeks before MS induction Treatment: 700 mg/Kg/day MO for 3 weeks after MS induction	Reduction in fasting glucose levels in preventive group compared with controls (80.09 ± 5.5 vs. 103 ± 3.8 mg/dL, *p* < 0.05); Reduction in OGTT in MS group compared with controls (12,616 ± 316.8 vs. 138,22.5 ± 213.93 mg/dL/120 min, *p* < 0.05).	No changes in treated rats	Significant reduction in triglyceride levels and in abdominal circumference.	[58]
Alloxan-induced diabetic mice	Leaf powder	Treatment time: 1, 3 and 5 h Diabetic Con: Untreated Diabetic positive Con: Insulin 0.7 IU/kg Diabetic MO: 100, 300, and 500 mg/kg MO	Reduction in diabetic rats at 5 h with 300 and 500 mg/kg MO (*p* < 0.01). 500 mg/kg dose presented significant BG reductions of 34.3%, 60.9%, and 66.4% after 1, 3, and 5 h, respectively.	No changes in diabetic mice	Significant increase in catalase, no changes in superoxide dismutase and significant reduction in MDA.	[59]
STZ-induced diabetic Wistar rats	Aqueous leaf extract	Treatment time: 3 weeks Con: Untreated Con MO: 100, 200, and 300 mg/kg MO Diabetic MO: 100, 200, and 300 mg/kg MO Diabetic positive Con: Glipizide 2.5 mg/kg	Significant reduction in fasting BG of diabetic rats treated with MO. Reduction after 1, 2, and 3 weeks with 200 mg was 25.9%, 53.5%, and 69.2%, respectively.	Not measured	Improvement of Hb and total protein levels.	[60]
Alloxan-induced diabetic Wistar rats	Aqueous leaf extract	Treatment time: 18 days Con: Untreated Diabetic Con: Untreated Con MO: 250 mg/kg MO Diabetic MO: 250 mg/kg MO	Reduction in diabetic rats. MO treatment reduced BG from 400 mg/dL to 200 mg/dL, *p* < 0.05.	Not measured	Significant reduction in triglycerides and MDA.	[10]
STZ-induced diabetic Sprague-Dawley rats	Aqueous leaf extract	Treatment time: 4 weeks Con: Untreated Con MO: 200 mg/kg MO Diabetic Con: Untreated Diabetic MO: 200 mg/kg MO	Reduction in BG of diabetic rats, from 266.50 ± 2.17 mg/dL to 148.83 ± 2.44 mg/dL after 200 mg of MO treatment, *p* < 0.001.	Not measured	Significant rescue of GSH and reduction of MDA.	[61]
Alloxan-induced diabetic Wistar rats	Aqueous leaf extract	Treatment time: 18 days Con: Untreated Diabetic Con: Untreated Con MO: 250 mg/kg MO Diabetic MO: 250 mg/kg MO	Reduction in diabetic rats treated with MO. Blood glucose levels lowered 3.6-fold, as compared with controls, *p* < 0.05.	Not measured	Normalization of SOD and catalase, and significant increase in GSH and reduction of MDA.	[62]
STZ-induced diabetic Wistar rats.High-fat diet induced diabetes C57BL/6 mice.	Aqueous leaf extract	Treatment time: 3 weeks Con: Untreated Con MO: 100 mg/kg (rats); 200 mg/kg (mice) Diabetic Con: Untreated Diabetic MO: 100 mg/kg (rats); 200 mg/kg (mice) Diabetic positive Con: metformin 42 mg/kg	Acute and chronic significant reduction in diabetic rats and mice. In rats, a reduction of 53.2% in fasting glucose after 4 h of oral administration of MO, whereas reduction of 41.7% was observed after 8 h, on day 1 and day 2, *p* < 0.05. In mice, a reduction of 34.23% on day 2, and 58.69% on day 3, *p* < 0.01.	Not measured	Improvement of hepatic functions. Significant increase in HDL. Significant decrease in cholesterol, VLDL, LDL, and triglycerides.	[63]
Alloxan-induced diabetic Wistar rats	Methanolic leaf extract	Treatment time: 6 weeks Con: Untreated Diabetic Con: Untreated Diabetic MO: 300 or 600 mg/kg MO Diabetic positive Con: metformin 100 mg/kg	Reduction in diabetic rats. BG was reduced by 76% at 300 mg/kg and 84% at 600 mg/kg, *p* < 0.001. In addition, glucose tolerance was improved by 56% and 57% with 300 or 600 mg/kg of MO, respectively, *p* < 0.001.	Significant increase in diabetic rats. Serum insulin levels increased 1.3–1.7-fold, *p* < 0.01.	Significant reductions in triglycerides, total cholesterol and LDL. Significant increase in HDL.	[64]
STZ-induced diabetic Wistar rats	Methanolic leaf extract	Treatment time: 6 weeks Con: Untreated Con MO: 250 mg/kg MO Diabetic Con: Untreated Diabetic MO: 250 mg/kg MO	Reduction in diabetic rats from 30.96 to 27.6 mmol/L, *p* < 0.05.	Not measured	Reduction in the activities of hepatic enzymes. Significant reduction of cholesterol, LDL, IL-6, TNF and MCP-1. Significant increase in HDL.	[65]
STZ-induced diabetic Wistar rats	Methanolic leaf extract	Treatment time: 3 weeks Con: Untreated Diabetic Con: Untreated Diabetic MO: 200 mg/kg/day MO	Reduction in BG levels in diabetic rats from 229 ± 9.05 mg/dL to 86 ± 4.2 mg/dL, *p* < 0.05.	Not measured	Oxidative stress attenuation and normalization of mitochondrial function in liver.	[66]
Alloxan-induced diabetic Wistar rats	Ethanolic leaf extract	Treatment time: 3 weeks Con: Untreated Diabetic Con: Untreated Diabetic positive Con: Sitagliptin 50 mg/kg Diabetic MO: 300 mg/kg MO Sitagliptin + MO: 50 mg/kg + 300 mg/kg MO	Acute but not chronic significant reduction in diabetic rats. The co-administration of sitagliptin and MO produced a decrease of 60% (90.00 ± 9.77 mg/dL, *p* < 0.01) in fasting BG after 2 weeks, as compared with day 1 levels (226.85 ± 21.81 mg/dl). No significant reduction at 4 weeks (38%, 138.57 ± 15.66 mg/dL).	No changes in diabetic rats	No changes in hyperglycemic retinopathy.	[67]
STZ-induced diabetic Long Evan rats	Ethanolic leaf extract	Treatment time: 120 min Diabetic Con: Untreated Diabetic MO: 250 mg/kg MO Diabetic positive Con: Glibenclamide 0.5 mg/kg	Reduction in diabetic rats (from ~6.5 to ~5.5 mmol/L, *p* < 0.05).	No changes in diabetic rats	-	[9]
C57BLKS/J Iar-+Lepr^db^/+Ledpr^db^ and C57BLKS/J Iar-m+/Lepr^db^ mice	Separate ethanolic extracts from leaves, seeds, and stem	Treatment time: 5 weeks Con: Untreated MO: 150 mg/kg MO Metformin: 150 mg/kg	Reduction in diabetic mice (only studied in leaves extract). Reduction in fasting BG from 483 to 312 mg/dL, *p* < 0.05.	Significant increase in diabetic mice (only studied in leaves extract). Increased insulin levels from 946 ± 92 to 1678 ± 268 pg/mL, *p* < 0.05.	Significant decrease in triglycerides and LDL. Decreased expression of inflammatory markers in the kidneys.	[68]
STZ-induced diabetic Albino rats	Seed powder	Treatment time: 4 weeks Con: Untreated Diabetic Con: Untreated Diabetic MO: 50 or 100 mg/kg MO for 4 weeks	Reduction in diabetic rats from 266 to 148 mg/dL, *p* < 0.05.	Not measured	Significant decrease in HbA_1C_. Significant reduction in lipid peroxide. Significant increase in antioxidant enzymes. Significant decrease in IL-6. Improvement of urinary and kidney functions.	[69]
C57BL/6J mice fed with very high-fat (VHF) or low-fat diet (LF)	Ethanolic Seeds extract	Treatment time: 12 weeks VHF: Untreated VHF + MO: MO mixed with ad libitum diet LF: Untreated LF + MO: MO mixed with ad libitum diet	Reduction in VHF-fed mice at 2 weeks from 500 to 250 ng/dL, *p* < 0.05.	Not measured	Reduced body weight, reduced inflammatory gene expression, increased antioxidant gene expression, and antibiotic-like restructuring of the gut microbiota.	[70]
STZ-induced diabetic ICR mice	Compounds extracted from seeds	Treatment time 2 weeks Con: Untreated Diabetic Con: Untreated Diabetic MO: 20 mg/kg per MO compound	Reduction in diabetic mice (*p* < 0.05).	Not measured	-	[71]
Alloxan-induced Swiss-Webster mice	N-hexane seeds extract	Treatment time: 8 days Con: Untreated Con MO: 40, 60, and 80 mg/kg MO Diabetic MO: 40, 60, and 80 mg/kg MO Diabetic beta-sitosterol: 18, 25, and 35 mg/kg	Significant reduction in diabetic mice (both in acute <6 h, and in subchronic treatment, up to 8 days). MO doses of 40, 60, and 80 mg/kg, shown a decrease of BG after 6 h of 49.2% (~130 mg/dL, *p* < 0.05), 53.4% (~115 mg/dL, *p* < 0.05), and 60.6% (~100 mg/dL, *p* < 0.05), respectively, as compared with controls (~240 mg/dL). Treatment with MO at day 8 showed BG reduction of 55.1% (~110 mg/dL, *p* < 0.05) with 40 mg/kg, 62.0% (~100 mg/dL) with 60 mg/kg, and 70.1% (~70 mg/dL, *p* < 0.05) with 80 mg/kg, as compared with controls (~250 mg/dL).	Significant increase in diabetic mice (8 weeks after subchronic MO treatment). Treatment with 40 mg/kg showed an increase to ~2.7 mg/L (*p* < 0.05), with 60 mg/kg to ~3 mg/L (*p* < 0.05), and with 80 mg/kg to ~3.7 mg/L (*p* < 0.05), compared with controls (1.5 mg/L).	Inhibition of alpha-glucosidase activity, increase in antioxidant activity, and reduction in HbA_1C_ levels. Improvement of diabetic neuropathy with significant decrease in tail-flick and hot plate latencies, and significant attenuation of static mechanical allodynia.	[72]
STZ-induced diabetic Wistar rats	Methanolic fruit extract	Treatment time: 3 weeks Con: Untreated Diabetic Con: Untreated Con + MO: 150 or 300 mg/kg MO Diabetic + MO: 150 or 300 mg/kg MO Diabetic positive Con: Glibenclamide 0.3 mg/kg	Reduction in diabetic rats treated with both 150 mg/kg MO (261.28 ± 13.20 to 213.37 ± 28.36 mg/dL, *p* < 0.05) and 300 mg/kg MO (261.8 ± 13.20 to 162.37 ± 22.78 mg/dL, *p* < 0.05).	Significant increase in diabetic rats treated with both 150 mg/kg MO (9.14 ± 1.11 to 12.21 ± 0.35 μU/mL, *p* < 0.05) and 300 mg/kg (9.14 ± 1.11 to 13.31 ± 0.78 μU/mL, *p* < 0.05).	Increases in protein levels, SOD, GSH, and catalase activity. Significant decrease in lipid peroxidation	[73]

Abbreviations: Blood glucose (BG); control (Con); experimental (Exp); glutathione (GSH); hemoglobin (Hb); high-density lipoprotein (HDL); low-density lipoprotein (LDL); malondialdehyde (MDA); metabolic syndrome (MS); monocyte chemoattractant protein-1 (MCP-1); oral glucose tolerance test (OGTT); superoxide dismutase (SOD); tumor necrosis factor (TNF); very low-density lipoprotein (VLDL).

**Table 2:** Evidence of the effects of MO treatment in human studies.

Condition (Type of Study)	Participants	MO Tree Part	MO Treatment (Duration)	Results of MO Treatment		Ref
				Blood Glucose	Other Effects	
Postmenopausal women (Randomized controlled trial)	30 females; age range 45–55 years	Leaf powder	7 g daily for 3 months	Reduction from a baseline of 125.6 ± 9.15 to 106.7 ± 7.23 mg/dL (*p* ≤ 0.01) in fasting BG levels.	Insulin not measured. Significant increase in Hb. Significant increase in antioxidant agents.	[77]
Healthy subjects (Quasi-experimental study)	5 females, 5 males; age range 20–40 years	Leaf powder	0 g (baseline), 1 g (first dose after 2 weeks), 2 g (second dose after 2 weeks) and 4 g (third dose after 2 weeks).	No significant changes.	Significant increase in plasma insulin 6 h after MO ingestion. Insulin levels with MO 0 g, 1 g, 2 g, and 4 g were 2.3 ± 0.9, 2.7 ± 1.0, 3.3 ± 1.4, and 4.1 ± 1.7 μU/mL, respectively (*p* = 0.024 and *p* = 0.011 for high dose vs. baseline and high dose vs. low dose, respectively). No changes in BUN, creatinine, ALT or AST.	[78]
Healthy subjects (Randomized crossover design study)	6 females, 4 males; age average 22.2 ± 0.2 years	Aqueous Leaf extract	500 mg on the first visit and 500 mg on the second visit, 2 weeks later.	No significant changes.	Insulin not measured.Significant increase in antioxidant capacity. Significant reduction in MDA.	[79]
Type 2 DM(Prospective randomized placebo-controlled study)	9 females, 7 males; age range 20–70 years	Leaf powder capsules	4 g daily before breakfast and dinner for 1 month.	No significant changes.	Insulin not measured. No significant difference in HbA_1C_. No changes in BUN, creatinine, ALT or AST.	[80]
DM (type not specified)(Prospective quasi experimental study)	48 females, 12 males; age range 19–65 years	Leaf powder capsules	500 mg capsule (3 times/day) for 12 weeks	Glycaemia not reported, but significant reduction in HbA1c in MO-treated patients.	Insulin not measured. Significant reduction in high specificity C-Reactive Protein, in MO-treated patients.	[81]
Type 2 DM and healthy subjects (Randomized controlled trial)	17 DM (9 females, 8 males); 10 healthy (6 females, 4 males)	Leaf powder	20 g once	Significant reduction in glycaemia up to 150 min after intake. The mean glycemic meal response was lower with 20 g MO (268 ± 18 mg/dL) than that obtained with Con (296 ± 17 mg/dL, *p* < 0.001) in diabetic subjects.	Insulin not measured. Significant reduction in α-amylase activity.	[82]

Abbreviations: Alanine aminotransferase (ALT); aspartate aminotransferase (AST); blood glucose (BG); blood urea nitrogen (BUN); control (Con); hemoglobin (Hb); malondialdehyde (MDA).
